# Efficacy of *Heterorhabdits indica* LPP35 against *Aedes aegypti* in domiciliary oviposition sites

**DOI:** 10.21307/jofnem-2019-050

**Published:** 2019-07-23

**Authors:** Bruna Silva, Alexandre M. Almeida, Claudia Dolinski, Ricardo M. Souza

**Affiliations:** Department of Entomology and Plant Pathology, Universidade Estadual do Norte Fluminense Darcy Ribeiro, Av. Alberto Lamego, 2000, Campos dos Goytacazes (RJ), Brazil.

**Keywords:** *Aedes aegypti*, Biological control, Dengue fever, Efficacy, Entomopathogenic nematode, *Heterorhabdits indica* LPP35, Mosquito

## Abstract

Entomopathogenic nematodes have been evaluated for control of mosquito species for decades. Depending on the nematode and mosquito involved, mortality rates of larvae (L) may reach 100% in vitro. Nonetheless, nematode efficacy at oviposition sites has rarely been assessed. *Heterorhabditis indica* LPP35 has been shown to kill over 75% of *Aedes aegypti* L3/L4 in cups and bottles outdoors. To assess its efficacy in indoor oviposition sites, different types/sizes of floor drains and pot saucers, and 65 liter water barrels, were infested with L3/L4 and treated with two doses of infective juveniles (IJs). In floor drains, mortality rates varied from 45 to 82%, with better results in the smallest drains. The adjustable dose of 25 IJs/cm^2^ of the drain’s bottom internal surface gave better results than the fixed dose of 100 IJs/larva. Mortality rates were only 28 to 53% and 0.1 to 1.7% in pot saucers and water barrels, respectively, probably because ridges and grooves that marked the bottom internal surface of these containers hindered the encounter of larvae and IJs.

The worldwide incidence, severity, and mortality rate of diseases caused by arboviruses – particularly dengue fever – have grown dramatically in recent decades (Anonymous, 2019). One recent estimate indicates 390 million infections/year, mostly in Central and South America, Africa and Asia (Bhatt et al., 2013). In the USA, 26 states are infested by *Aedes aegypti* L. (Diptera: Culicidae), the main vector of dengue fever. Hawaii and Puerto Rico have faced recent outbreaks of this disease, while many locally transmitted cases have occurred in Europe (Calzolari, 2016; Hahn et al., 2016).

The main strategies to combat *A. aegypti* are urban habitat management, to minimize oviposition sites, and insecticide application. Insecticide resistance has increased rapidly in recent years (Moyes et al., 2017), making the search for alternatives a global priority. Larval control, in domiciliary and public spaces, is a major challenge (Roiz et al., 2018).

For larval control, greater focus has been placed on chemical and microbial larvicides, insect growth-regulators and predatory fish, copepods and Toxorhynchite larvae. Nonetheless, field trials have brought mixed reports of the prospect of these approaches to prevent or curb dengue fever outbreaks (Horstick and Runge-Ranzinger, 2018; Achee et al., 2019).

Recent reviews on biological control of mosquitoes (Benelli et al., 2016; Huang et al., 2017) have not even mentioned entomopathogenic nematodes (EPNs) as potential agents. Nonetheless, a range of *Heterorhabditis* and *Steinernema* species have been shown to kill, at different rates, larvae and pupae of several mosquito species. Early studies indicated that mosquito larval stages 3 and 4 (L3, L4) readily ingest EPN infective juveniles (IJs). Most IJs are injured by larval mouthparts, but some enter the hemocoel and overcome the insect’s defenses, causing mortality (Daad, 1971; Poinar and Kaul, 1982; Molta and Hominick, 1989).

Most studies on the efficacy of EPNs against mosquito larvae have been conducted only in the laboratory, with a variety of methodological procedures that limits generalizations (for a review see Cardoso et al., 2015). Recent assays still have not examined key parameters that may determine the efficacy and viability of EPNs for biocontrol of mosquitoes (Chaudhary et al., 2017; Ulvedal et al., 2017; Toksoz and Saruhan, 2018; Dilipkumar et al., 2019).

*Heterorhabditis indica* Poinar, Karunakar, and David strain LPP35 was serendipitously found during a survey of nematodes in tank-forming bromeliads, a freshwater ecosystem abundant in culicids (Robaina et al., 2015). Among different EPNs tested, *H. indica* LPP35 stood out with efficacy of 80 to 96% at killing L3/L4 larvae of *A. aegypti*. Those results were obtained with a dose as low as 100 IJs/larva (Cardoso et al., 2015).

In a follow-up study, the efficacy of *H. indica* LPP35 was assessed outdoors, in plastic cups, bottles, and buckets, which mimicked typical oviposition sites. The efficacy was above 75% in cups and bottles, but fell to 40% in buckets (Cardoso et al. 2016). The authors concluded that the key parameters for *H. indica* LPP35 efficacy against larval stages of *A. aegypti* are: (i) the dose of IJs/larva, which should be at least 80 to 100; and (ii) the dose of IJs/cm^2^ of the bottom internal surface of the oviposition site. This is important because mosquito larvae graze at the bottom of oviposition sites, where EPNs remain after application. Since larvae must ingest several EPNs to get infected, about 6.5 IJs/cm^2^ resulted in a mortality rate around 80%, while at 25 IJs/cm^2^ it reached 100%.

Urban populations of *A. aegypti* lay eggs primarily in intra- or peri-domiciliary sites, such as pot saucers, floor drains, and in neighborhoods not served by piped water, water barrels (Powell and Tabachnick, 2013). In pot saucers, the standing water may evaporate within hours, while floor drains and water barrels are perennial sites putatively favorable to EPN’s survival and action. These three kinds of sites also differ in shape and square area of the bottom internal surface. Also, floor drains have a smooth internal surface, while pot saucers and water barrels usually have grooves and ridges. These differences may interfere with the efficacy of EPNs against mosquito larvae.

Hence, the goal of this work was to assess the efficacy of *H. indica* LPP35 in the main domiciliary sites chosen by *A. aegypti* females to lay eggs – floor drains, pot saucers, and water barrels.

## Material and methods

### Nematode and mosquito culturing

Larvae of the wax moth (*Galleria mellonela* L.) were infected with *H. indica* LPP35 in 9 cm Petri dishes. About seven days later, the larvae were transferred to White traps for extraction of IJs. Infective juveniles were maintained in culture flasks with distilled water, in the dark, at 16°C, for no longer than seven days before being used in the assays. Eggs of *A. aegypti* (Rockefeller strain) were placed in trays with filtered tap water, in the dark, at 25°C, for eclosion. After hatching, a small amount of mouse feed was added to the trays to feed the larvae. Stages L3/L4 were individually picked with a Pasteur pipette and used in the assays.

### Assays in floor drains

**Figure 1:**
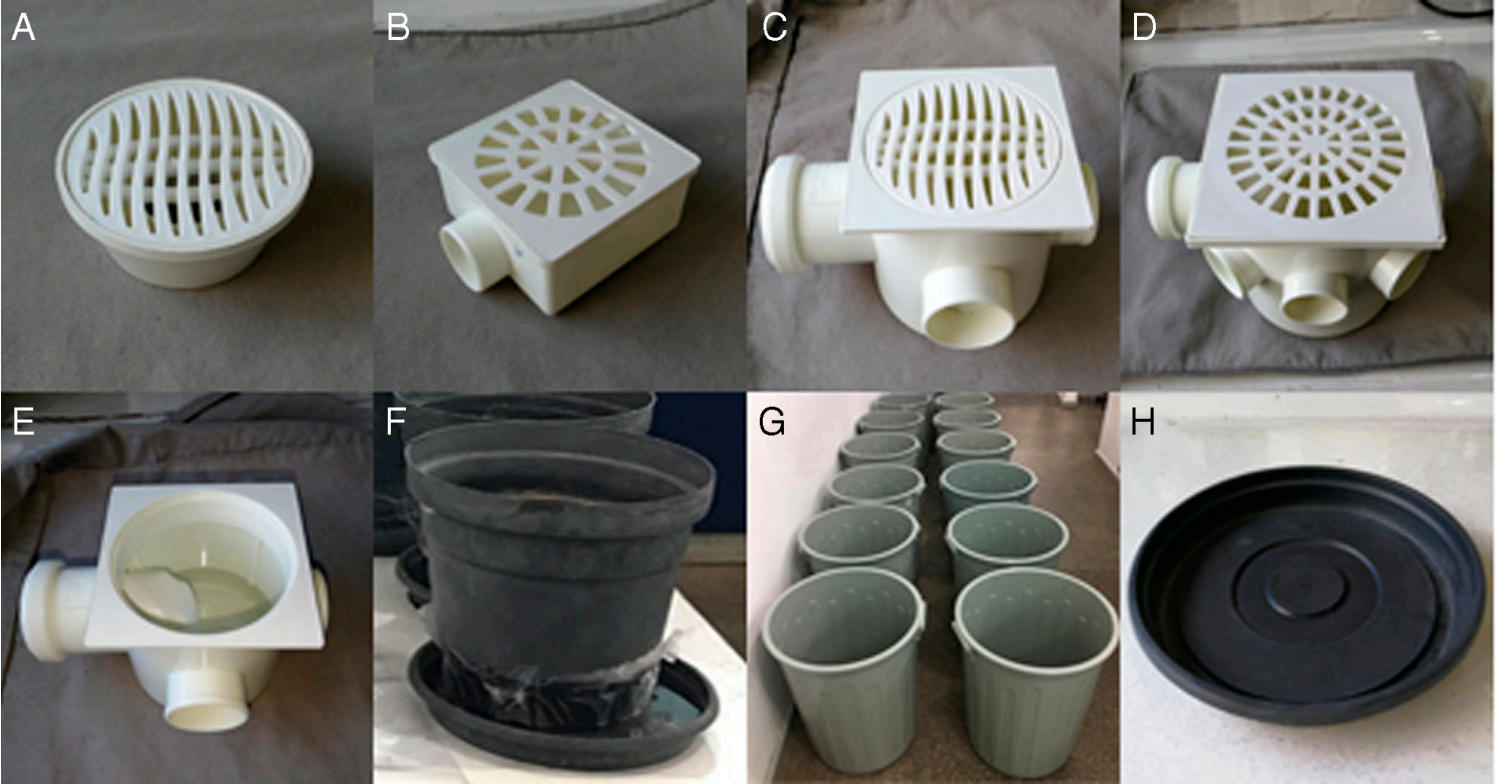
(A–D) floor drain types (1–4); (E) water permanently retained in the bottom of all drain types; (F) medium size pot and saucer; (G) 65 liter water barrels; (H) ridges and grooves on the surface of pot saucers.

Floor drain types 1 through 4 were used (Fig. 1A–D). Type 1 have a bottom internal surface area of 34.5 cm^2^. In type 1, the water permanently retained in the drain’s bottom (Fig. 1E) is about 40 ml. In types 2 through 4, the bottom internal surface areas are 47.7, 83.3, and 188.5 cm², respectively, and the amounts of water retained are 110, 270, and 1,120 ml, respectively.

For each drain type, 10 drains received 10 L3/L4 and 1,000 IJs. The blank control consisted of 10 drains in which L3/L4 only were applied. The drains were placed in a growth chamber, in the dark, at 25°C. A voile fabric was attached to the drain to avoid releasing adult mosquitoes into the environment. The evaluation was carried out 7 to 8 d later, when adult mosquitoes had emerged in the blank control. Dead larvae and pupae were counted and inspected for the presence of nematodes in their body. This assay was repeated twice.

A second set of floor drain assays was conducted as described before, with the exception of using the adjustable dose of 25 IJs/cm^2^ of drain bottom internal surface. This assay was repeated once.

### Assays in pot saucers

Small, medium (Fig. 1F), and large pots with saucers were used. When tap water was poured in the pot, it leaked through the bottom holes and created the saucer’s standing water. For the small, medium, and large saucers, the water volumes were 20, 65, and 368 ml, respectively. These volumes were retained mainly around the pot, in areas of 13.3, 87.9, and 245 cm², respectively.

For each saucer size, the assay procedures were as described before, except that the pots and saucers were maintained on laboratory benches, at 25°C. The assays, using either 1,000 IJs/saucer or the adjustable IJ dose, were conducted twice.

### Assays in water barrels

65 liter barrels were used, with a bottom internal surface of 961 cm^2^. Sixty liters of tap water was added, with the water column reaching 50 cm high. Ten barrels received 10 L3/L4 and 25 IJs/cm^2^ (24,000 IJs), and 10 barrels served as blank controls, receiving 10 L3/L4 only. The assay procedures were as described before, except that the barrels were kept closed with their own lids on the laboratory floor, at 25°C. This assay was repeated once.

### Data availability and analysis

The assays’ raw data are publicly available at: https://figshare.com/articles/Silva_et_al_Raw_Data/8320595.

For all assays, the data on the dead mosquito larvae were tested for homogeneity of variances (Cochran and Bartlett tests) and for normality of errors (Lilliefors test), at 5% probability (Ribeiro, 2001). Since the assumptions were satisfied, ANOVA was conducted considering time as one of the factors. No statistical significance was found for the floor drain assays, so their data were pooled. Time was a significant factor for assays in pot saucers and water barrels, so the data were analyzed separately for each assay. The treatments’ mean numbers of dead larvae were compared through the Tukey test at 5% probability, and expressed in the tables as mortality rate (%).

## Results and discussion

**Figure 2:**
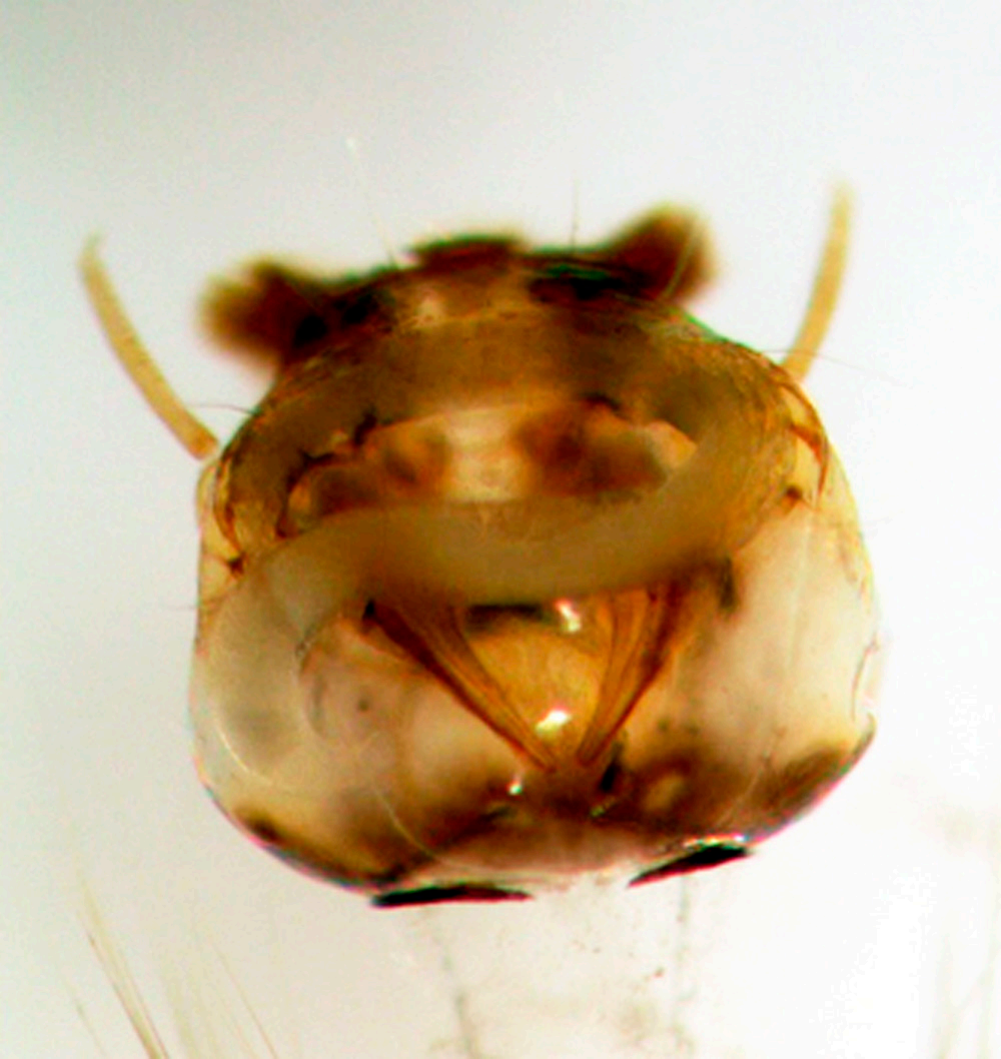
Parthenogenetic female of *Heterorhabditis indica* LPP35 inside the head of an *Aedes aegypti* larvae.

*Heterorhabditis indica* LPP35 is a promising agent for *A. aegypti* larval control in small, cryptic domiciliary environments. When floor drains were treated with 1,000 IJs, the mean mortality rate reached 74% in the smallest drain type, and progressively fell to 45% in the largest one (Table 1). Most nematodes were seen in the larval thorax, but occasionally also in the head (Fig. 2). In a second set of assays using the IJ dose suggested by Cardoso et al. (2016) – 25 IJs/cm^2^ – the mortality rate peaked at 82%. The nematode efficacy gradually declined to 56% in the largest drains.

**Table 1.** Mortality rate (%) of *Aedes aegypti* L3/L4 in four types of floor drains, seven days after treatment with *Heterorhabditis indica* LPP35 infective juveniles (IJs), at two distinct doses.

Types (bottom internal surface area, in cm^2^)			
Treatments	1 (34.5)	2 (47.7)	3 (83.3)	4 (188.5)
*100 IJs/larva*				
Treated	74a	67a	64a	45a
Control	23b	23b	10b	0b
*25 IJs/cm* ^*2*^				
Treated	82a	78a	76a	56a
Control	3.5b	3.5b	5b	1b

Notes: Values are means of three assays, each with 10 drains/type, and 10 larvae/drain. In the columns, values followed by different letters are statistically different according to the Tukey test at 5%.

This suggests that each mosquito larva browses a certain bottom area of the oviposition site, and that at 25 IJs/cm^2^ most larvae ingest enough nematodes to get infected and killed. Nonetheless, it remains unclear why nematode efficacy declined in the largest drains, since the IJ dose was the same in all sizes. It is possible that with more nematodes applied in the largest drains, they formed more and larger clumps, as typically occur with some *Heterorhabditis* species when maintained in water. This would make encounters between the larvae and nematodes less likely.

In pot saucers, applying 10 IJs/mosquito larva resulted in mortality rates of 46 to 53% in the smallest saucers, decreasing to 35% in the largest ones (Table 2). The IJ dose of 25 IJs/cm^2^ did not improve the efficacy, even when the IJs/mosquito larva rate was about 600. The bottom internal surface of pot saucers, marked with grooves and ridges (Fig. 1H), may have hindered the encounter of mosquito larvae and nematodes. Finney and Harding (1981) reported a steep decline in the efficacy of *S. carpocapsae* in causing mortality of *A. aegypti* in containers filled at the bottom with sand or leaves, which also hindered the encounter of larvae and nematodes.

**Table 2.** Mortality rate (%) of *Aedes aegypti* L3/L4 in pot saucers, seven days after treatment with *Heterorhabditis indica* LPP35 infective juveniles (IJs), at two distinct doses.

Sizes (bottom internal surface area, in cm^2^)		
Treatments	Small (13.3)	Medium (87.9)	Large (245)
*100 IJs/larva*			
Assay 1			
Treated	46a	29a	35a
Control	3b	11b	14b
Assay 2			
Treated	53a	53a	35a
Control	18b	13b	8b
*25 IJs/cm* ^*2*^			
Assay 1			
Treated	31a	44a	38a
Control	0b	0b	0b
Assay 2			
Treated	28a	37a	30a
Control	0b	0b	0b

Notes: Values are means of 10 pot saucers/size, and 10 larvae/pot saucer. In the columns, values followed by different letters are statistically different according to the Tukey test at 5%.

Grooves and ridges were also present in the bottom internal surface of the water barrels. The dose of 25 IJs/cm^2^ resulted in 24,000 IJs applied/barrel, but the efficacy was unacceptably low: mortality rates of 0.1 and 1.7% in assays 1 and 2, respectively. This clearly suggests that a much higher dose would be needed to treat water barrels, and that EPNs would not be feasible to treat abandoned swimming pools, which are also important domiciliary oviposition sites.

Since Welch (1960) (cited by Finney and Harding, 1981), EPNs have been investigated for biocontrol of various mosquito species. In some *in vitro* assays, mortality rates were high, with LC99 being reached with doses as low as 170 IJs/larva (e.g. Poinar and Kaul, 1982; Zohdy et al., 2013; Chaudhary et al., 2017). Nonetheless, few studies have tested EPNs in semi-field or field conditions, or investigated micro-environmental factors that might affect EPNs’ efficacy. For instance, although larvae of the black fly (*Simulium vittatum*) were readily killed by *S. carpocapsae in vitro*, this was ineffective when applied in streams (Gaugler et al., 1983). In bromeliads, Cardoso et al. (2016) reported a mortality rate of *A. aegypti* larvae of only 23%, probably because most IJs fell into the plant leaf axils, away from grazing larvae. Reports such as these should not come as a surprise, since a range of abiotic factors have been shown to affect EPN efficacy, at least in agricultural settings (Shapiro-Ilan and Dolinski, 2015).

In this study, *H. indica* LPP35 was particularly efficient in floor drains, with an indication that doses above 25 IJs/cm^2^ should be applied. Peri-domiciliary oviposition sites, such as pot saucers and large water containers, seem not to be appropriate for EPNs. These sites are more prone to sun exposure, heating of the water retained, and desiccation.

Although EPNs seem promising to control *A. aegypti* in just one subset of oviposition sites, this should not discourage further studies, particularly on the matters of doses and application technology. The control of *A. aegypti* has become a daunting task worldwide, since it requires efficient methods, good local governance, and community engagement. A new concept of mosquito control has emerged, which involves the development of approaches that are complementary, niche strategies, rather than expecting one approach to become the default intervention across a wide range of settings (Achee et al., 2019). Further studies may lead EPNs to become one more option for larval control of *A. aegypti* in domiciliary cryptic oviposition sites.
